# Investigating Effect of Service Encounter, Value, and Satisfaction on Word of Mouth: An Outpatient Service Context

**DOI:** 10.3390/ijerph15010132

**Published:** 2018-01-13

**Authors:** Li-Chun Hsu

**Affiliations:** Department of Cultural Resources and Leisure Industries, National Taitung University, 369, Sec. 2, University Rd., Taitung 95092, Taiwan; lchsu@nttu.edu.tw; Tel.: +886-912-227-792

**Keywords:** service encounters, service value, satisfaction, word of mouth (WOM), sustainability medical services

## Abstract

This study investigates the relationships among service encounter, service value, patient satisfaction, and word-of-mouth (WOM) intention from the viewpoint of interactive marketing. Data were collected using a questionnaire survey. A total of 372 questionnaires were obtained and 350 of these questionnaires were valid (94.09%), and a structural equation model was used to analyze the data. This study proposed seven hypotheses, and five of the seven hypotheses were supported. Service encounters indirectly affect their patient WOM through service value and satisfaction. Therefore, service value and satisfaction play a crucial mediating role in linking service encounters and WOM. This study determined WOM intentions in an outpatient service context and provides crucial business implications for teaching hospitals to enable them to improve their service quality and achieve a sustainable operation.

## 1. Introduction

The interactive relationship between service providers and customers during service encounters has attracted attention over the past few years in the marketing and management literature. Healthcare industries in developing countries such as India are growing at a relatively fast pace with a higher demand for services from both foreign and local patients [1]. However, people in Taiwan tend not to assess the relationship between service encounters of outpatients and other variables. Since the 1990s, customer orientation has become a crucial concept in the marketing field. The Taiwan healthcare system enables healthcare practitioners to provide excellent service quality and establish long-term and favorable relationships with their patients through service encounters, which are thus prerequisites for success. Although the national health insurance program is being reformed continuously and the medical environment is transforming rapidly, the medical market in Taiwan has become highly competitive over time. Hence, the medical industry began valuing the importance of the service management concept. The management concept of medical institutions has since been transformed into providing a healthcare “service encounter” to encourage patients to engage in word-of-mouth (WOM) communication. Thus, present medical institutions are no longer confined to providing only medical treatment, but also ensuring that customers enjoy the comfort and protection of medical professional services [2]. In the professional service area, placing attention on medical care is imperative. In recent years, because of advancements in public health medicine and higher education levels, the national health standard in Taiwan has generally improved. A service recipient assesses the service quality on the basis of the actual service and interaction with the service provider. Interaction with a service provider is a crucial element that influences overall patient satisfaction. In common service encounter situations with consumers, professional service providers focus on effectively delivering services. Providers also expect to gain the trust of a service recipient, which induces the recipient to recommend the service to other consumers for sustainable patronage. Therefore, in the course of service delivery, face-to-face interaction between service providers and outpatients creates positive consumer perceptions toward the service quality provided and enables proper planning and appropriate guidance regarding the customers’ perceptual judgment. These service encounters are also a favorable strategy for any marketing recommendation or endeavor.

Li and Yuan [3] reported that using a customer (patients) relationship network in WOM marketing is crucial for gaining an advantageous position in a fiercely competitive market. Hospitals should effectively capitalize on the use of a customer (patient) relationship network to generate positive effects that advertisements cannot produce. From a medical service encounter perspective, the present study examined the differences in patients’ considerations of doctor treatment and the physical environment; specifically, this study investigated how patients’ considerations affect their satisfaction with treatment services and how a local medical practitioner uses basic medical resources to create a unique WOM recommendation for inducing patients to visit the medical service again.

This study contributes to the health care and hospitals literature in several ways. First, it shows how service encounters (i.e., service personnel and facilities) influence outpatients’ WOM in hospitals. Second, in addition to supporting previous studies that have shown that service value and satisfaction factors are important in regard to predicting WOM in health care systems, this study shows how service value mediates the effect of service encounters on outpatients’ WOM.

On the basis of the research motivations, the objectives of this study are descried as follows. First, to examine the determinants of outpatient WOM regarding service encounter approaches for identifying which approach acts as the decisive factor influencing outpatients to maintain a favorable service relationship (e.g., service value and satisfaction) with a hospital; second, to identify factors influencing the intention of outpatients to generate WOM recommendations.

## 2. Research Model, Literature, and Hypotheses

### 2.1. Research Model

**Figure 1:**
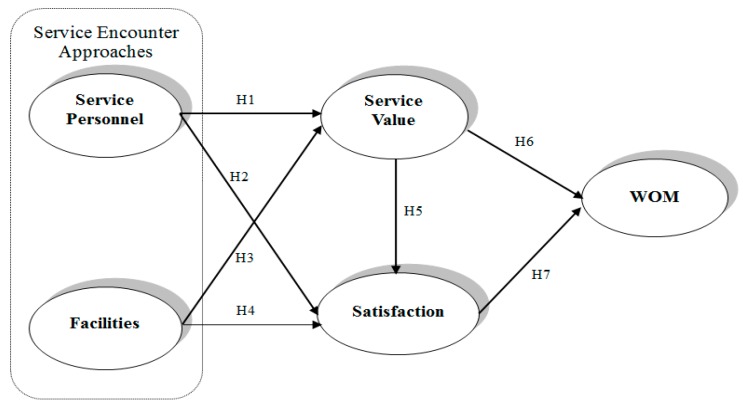
Proposed model.

This study further explores outpatients’ perceptions of satisfaction with service quality and how these affect their subsequent WOM intentions on the basis of service encounter approaches. Figure 1 shows the proposed model.

### 2.2. Service Encounters

Service encounter is considered the core of any service marketing issue and considerably affects service quality control, service delivery systems, and customer satisfaction [4]. Solomon et al. [5] devised the service encounter concept and defined it as a face-to-face interaction between service providers and recipients during the process of service consumption. Shostack [6] offered a broader definition of this concept: a service encounter is “a period of time during which a consumer interacts with a service”. Service encounter is generally defined as a consumer’s direct contact with a service provider, including both face-to-face interaction and experience [7]. Voorhees et al. [8] defines service encounter as “any discrete interaction between the customer and the service provider relevant to a core service offering”. Moreover, according to experience, the effect of the physical environment on behavior and image creation is particularly crucial for service industries, such as hotels, retail establishments, and the healthcare industry [9,10].

### 2.3. Service Value

The notion of value, at least from a marketing approach, has a clear subjective orientation, with most authors attributing an evaluative judgment to the value aspect [11,12,13,14,15]. In a service context, value can be understood as a conception that involves a trade-off between what a customer gains from the service and what the customer offers to obtain it. In short, a customer is presumed to make a purchase decision on the basis of “value”, which also underlies the notion of “cost”. Service value is conceptualized as the consumer evaluation of the utility of perceived benefits and sacrifices. Thus, consumers may cognitively integrate their perceptions of what they gain (i.e., benefits) versus what they have to give up (i.e., sacrifices) to receive the services.

### 2.4. Satisfaction

Satisfaction is widely used as and considered a behavioral indicator. The first study on the origin of customer satisfaction was conducted in 1965 and proposed by Cardozo [16]. Numerous scholars have interpreted and defined satisfaction from various viewpoints. Satisfaction is defined as a personal, subjective evaluation of care. The American Pain Society Quality of Care Committee [17] reported that satisfaction ratings reflect patient evaluations of their satisfaction with what has happened. Satisfaction is broadly defined as a consumer response to the evaluation of the perceived discrepancy between prior expectations and actual product performance as perceived after consumption [18,19,20]. Petruzellis et al. [21] agreed that customers are satisfied when a service fits their expectations, very satisfied when the service exceeds their expectations, and completely satisfied when they receive more than they originally expected. Customer satisfaction is a tool that can be used to measure customer feelings and understand customer needs and expectations.

### 2.5. WOM

In the past 50 years, numerous scholars have argued that word-of-mouth (WOM) was a crucial research issue [22,23,24]. Arndt [22] reported that WOM is a “marketplace information dissemination mechanism in which customer opinions concerning identified enterprises, products/services, and consumer experiences are communicated through informal interpersonal interactions”. Word-of-mouth is defined as verbal, informal, person to person communication between a perceived noncommercial communicator [25,26]. Therefore, WOM is the process of talking about brands, products, or services and occurs between receivers and communicators without real business intent. WOM refers to the communication of product information between two persons who are non-marketing personnel [27]. Therefore, applying the WOM effect is a more effective marketing approach than implementing traditional marketing.

### 2.6. Research Hypotheses

Empirical studies have not examined the relationship between service encounter and value. However, a review of indirect research and related literature revealed that service encounter and value are related [13]. For example, service personnel, in the process of service interaction with outpatients, and a favorable or unfavorable surrounding environment can show outpatients the value of feelings. Thus, value is not only a measurement of price, but also that of outpatient feelings regarding the effectiveness of services provided by service personnel and medical facilities.

During the service encounter process, service personnel and facilities may be precursors influencing consumers’ satisfaction with the service and enhancement of value. Hsu [28] conducted an empirical study on other industries, which addressed service quality, and a satisfaction survey in international tourist hotels in Taiwan, and reported that the attitude of staff and hotel equipment does affect customer satisfaction and repeat intention. In their healthcare system research, Chang et al. [29] examined the influence of service encounter and patient satisfaction, and the results verified that the medical staff, nursing staff, and service personnel, as well as the space facilities, are prefactors that effectively forecast medical treatment satisfaction. Thus, the two factors are correlated; that is, they measure service encounter (service personnel and physical facilities) on the basis of outpatient satisfaction and service value, which then act as a basis for hospital service quality improvement. Specifically, diversified and differentiated services can directly affect customer satisfaction [30]. Lee et al. [31] also indicated that the quality of tangibles must be examined as a direct preference driver for customer satisfaction. The service being studied involves different interactions for each episode. Thus, direct effects of customer perceptions of specific interactions on service value and satisfaction seem reasonable [13]. This approach can facilitate improving the quality of hospital services. Thus, this study proposes hypotheses **H1**–**H4**.

**Hypotheses 1** **(H1).** Hospital service personnel significantly and positively affect service value.

**Hypotheses 2** **(H2).** Hospital service personnel significantly and positively affect satisfaction.

**Hypotheses 3** **(H3).** Hospital facilities significantly and positively affect service value.

**Hypotheses 4** **(H4).** Hospital facilities significantly and positively affect satisfaction.

Regarding the association between service value and satisfaction, Fornell [32] defined perceived quality as the first element of overall service satisfaction and perceived value as the second element. Empirical studies have reported that perceived value positively affects satisfaction in the service industry [33]. Although perceived value occurs at different stages of a purchase process, satisfaction is universally included as a postuse or postpurchase evaluation. This finding clearly introduces a causal order through which satisfaction is understood as the result of the perception of value, as demonstrated by various studies, including Fornell et al., Oh, Caruana et al., Babin and Kim, and Gallarza and Gil [32,34,35,36,37,38]. Therefore, this study proposes the following hypothesis:

**Hypotheses 5** **(H5).** Hospital service value significantly and positively affects satisfaction.

Regarding WOM intention, Li and Yuan [3] reported that a patient relationship network is key to ensuring that a hospital gains a competitive advantage and crucial for success. Although this study did not provide much evidence on the relationships among service value, satisfaction, and WOM, WOM is a type of loyalty. Service firms share the same interest in service encounters as customers and employees do. Favorable encounters may provide service firms with a competitive edge and may result in repeat purchases and positive WOM [39]. According to Garbarino and Johnson and Athanassopoulos [40,41], loyalty is positively correlated with repurchase intention. These studies have also revealed that patient satisfaction and word-of-mouth are correlated in the medical service industry.

Several studies have been conducted on the consequences of satisfaction evaluation. The findings of such studies have strongly demonstrated that customer satisfaction positively affects positive WOM [42,43]. Wu and Yang [44] reported that customer satisfaction affects WOM propagation in the movie industry. Cristiane and Daniel [45] proposed the existence of an antecedent of consumer loyalty, namely the perceived value level, which concerns consumer evaluation regarding the benefits and costs of maintaining a relationship with a company [46]. The logic here is that if maintenance costs are low, even when a consumer extracts benefits from relational exchanges with a company, that relationship becomes highly attractive, increasing the consumer’s display of loyalty, thereby explaining the positive WOM intention [46]. Cristiane and Daniel [45] proposed that perceived value emerges as a significant antecedent of consumer WOM and patient satisfaction in generating more customer loyalty in hospitals [1]. The current study thus proposes **H6** and **H7**.

**Hypotheses 6** **(H6).** Hospital service value significantly and positively affects WOM.

**Hypotheses 7** **(H7).** Hospital satisfaction significantly and positively affects WOM.

## 3. Methodology

As illustrated in Figure 1, the research structure was established based on the aforementioned literature review and assumptions. In the following sections, we explain the data collection procedures and operationalization of the five constructs. We then present descriptive statistics and the selected observable indicators measured by the constructs. Subsequently, we present the empirical test of the hypotheses conducted using the linear structural relationship (LISREL) 8.51 technique proposed by Jöreskog and Sörbom [47]. The test was conducted in two steps. The first step involved running a measurement model test, which secured the construct and discriminant validity. The second involved testing the hypothesized relationships in accordance with the model (Figure 1). On this basis, we present the resulting structural model. The significance of the resulting model was evaluated and used for possible verification of the relationships among the five constructs, thereby testing the hypotheses.

### 3.1. Sample and Data Collection

A questionnaire survey was used to collect data from the outpatients of a regional teaching hospital in Yi-Lan, Taiwan. A total of 372 questionnaires were collected from the survey respondents. After 22 invalid questionnaires (e.g., because of inconsistent responses) were discarded, 350 questionnaires were statistically analyzed. To ensure flexibility and a high response rate in the survey, data were collected through face-to-face interviews that involved written questionnaires. The study focused on outpatients receiving medical service, and these outpatients were surveyed in each clinic waiting area. The respondents that had just experienced their medical service process in the first step were asked to response specifically. The interviewer provided the assistance or explanations to the respondent if questionnaire items were not clear. Finally, after completing the questionnaire, the respondents were thanked and given a symbolic gift as a token of appreciation.

### 3.2. Measurement Development

All measures were obtained from past studies and modified to fit the specific hospital service context. As a measure of service encounter, we adopted a two-dimensional (service personnel and facilities), seven-item scale named SP (service personnel), which was originally developed by Parasuraman et al. and Winsted [48,49], and an eight-item scale named FAC (facilities), which was originally developed by Bitner [50]. Service value was measured using scaled items adopted by Yoon, Choi, and Park [51], who constructed four-item scales asking questions on whether consumers appraise the service value considering the costs (money, time, efforts, and energy) incurred in obtaining the services they wanted. Satisfaction was measured using scaled items adopted by Gronholdt and Martensen [52]. A five-point Likert-type scale was used for this study. The WOM recommendation intention was measured using scaled items adopted by Maxham [53]. Multivariate items were employed on a five-point Likert-type scale for two dimensions. Each item had a scale ranging from 1 (I do not agree at all) to 5 (I totally agree). The questionnaire was prepared in English, translated into Chinese by independent translators, and then translated back into English to ensure its accuracy and consistency with appropriate guidelines [54]. Before the formal questionnaire was administered, a two-step semantic amendment was conducted to fit the context of this specific hospital. The first step was to select 30 respondents for conducting semantic reconfirmation, and the second step was to deliver a small-scale survey (60 samples) to amend the context and confirm the final questionnaire items and semantics.

To determine whether differences existed in the responses (i.e., bias) of the morning, afternoon, and night respondents, an independent sample *t* test was applied to the five numerical data variables, and no statistically significant differences at *p* < 0.05 were observed. Chi-square analyses were applied to five nominal data variables comparing the morning, afternoon, and night respondents, and no statistically significant associations at *p* < 0.05 were observed. In addition, common method variance (CMV) as a single-source bias, can lead respondents with consistent responses to generate CMV problems, especially if samples are from a single source measurement [55]. In order to avoid sample bias, this study examined whether there were CMV problems by using Harman’s one-factor-test [56], which is mainly used for exploratory factor analysis (EFA). This study conducted EFA analysis with all items and extracted six factors. The explained variance proportion by the first factor was 40.25%, which was lower than 50%.

## 4. Results

### 4.1. Demographic Characteristics

Most of the respondents were females (63.1% of the total sample), 28.9% were aged 30–39 years, and for 83.1%, this was not their first medical treatment. In the outpatient division, outpatients were general internal medicine outpatients (71 [20.3%]), followed by gastrointestinal, family medicine, general surgery, obstetrics and gynecology, and pediatrics patients (34 [9.6%]). The outpatient clinics were busiest in the afternoon hours (168 [48%]), followed by morning hours (117 [33.4%]), and then night hours (65 [18.6%]). The registered mode of “register locality on that day” was the highest (129 [36.9%]), followed by “locality” (82 [23.4%]). The outpatient ratio was more than half of the locality or registered on-the-spot appointments. Among the hospital selection reasons, “close to house” corresponded to a considerable proportion of the respondents (136 [29.2%]), followed by “traffic convenience” (86 [18.5%]). Table 1 shows the demographic characteristics of the respondents.

### 4.2. Data Analysis

Several statistical techniques, including a reliability and validity analysis (confirmatory factor analysis, CFA), correlation analysis, and LISREL, were used in this study. To test the model and the hypotheses, structural equation modeling was used. This study applies the two-stage approach suggested by Anderson and Gerbing [57]. The first step in this approach involves testing the measurement model using CFA, and the second step involves testing a series of structural models, including the hypothesized model. A measurement model describes how well the observed or measured variables (indicators) serve as measurement instruments for the underlying latent variables. The measurement model also estimates nondirectional relationships (correlations) among the latent variables. The composite reliability and average variance extracted met the criteria proposed by Gaski and Nevin [58], indicating high convergent validity [59]. Table 2 shows the data that demonstrated high convergence. The square root of the average variance extracted in each construct exceeded the coefficient of correlation of that particular construct with other constructs, confirming discriminant validity [58,60]. Each of the coefficients representing the correlations among the constructs was significant and met the discriminant validity criteria (Table 3). Both Anderson and Gerbing and Gefen, Straub, and Boudreau [57,61] have recommended assessing data by using a model fit index as the measurement model. The measurement model exhibited an adequate fit: χ^2^/df = 2.042, comparative fit index = 0.919, goodness of fit index = 0.912, adjusted goodness of fit index = 0.923, and root mean square error of approximation (RMSEA) = 0.043 (Table 4). According to the evaluation criteria of [62], the measurement model demonstrated adequate fit.

### 4.3. Model Estimate and Modification Indices

A structural model tests a general model that prescribes the relationships among the latent variables. In this study, a maximum likelihood estimate was applied to the latent variables for determining the path coefficient values of all the variables. Table 5 lists the estimated value of the parameter and test results for analyzing seven path coefficients in this study. All the hypotheses were supported, except H_4_. This path indicates that hospital facilities do not significantly and positively affect satisfaction.

The path indicates that hospital facilities do not significantly and positively affect satisfaction, and the RMSEA value of the model (0.088) was higher than 0.08 (RMSEA value of 0.05–0.08 indicates an optimal fit). In addition, the values of the modification indices revealed that to model the relationship between a few latent variables and the observation variable, revising the structure of the new model and searching for an optimal adjustment is necessary.

**Figure 2:**
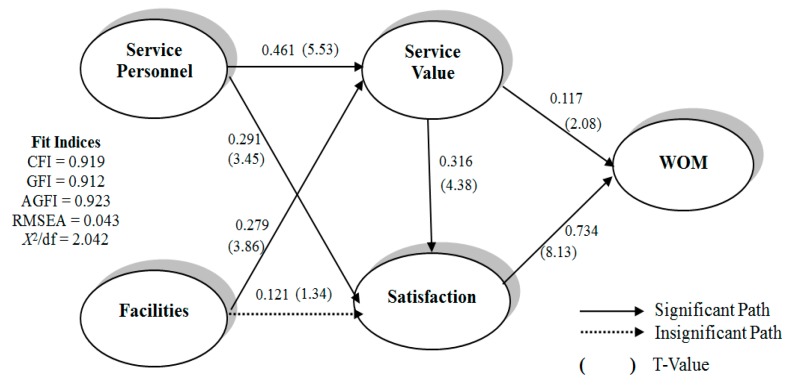
Structural model. * Values denote path standard solutions and T value (within parentheses).

Table 5 shows the path coefficient and relationship between the cause and effect (Figure 2), which was affected by outpatients’ perceptions of service value as service personnel and facilities during the service encounter. Therefore, the effect of service personnel (path coefficient: 0.461) is higher than that of facilities (path coefficient: 0.279). Satisfaction was affected by service personnel in the service encounter. Furthermore, facilities indirectly strengthened and improved satisfaction through service value. Overall, to improve patient service value, satisfaction and WOM intention must be improved. One of the WOM intentions is satisfaction, which was the most affected (path coefficient: 0.734). Thus, satisfaction was the main factor affecting WOM intention (path coefficient: 0.734). Thus, satisfaction was clearly a crucial determinant of the structure of this study model. Deleting this path and re-estimating the model did not significantly change the model fit. Table 5 shows the estimates for this trimmed model.

The results indicated that service facilities did not significantly and positively affect satisfaction; hence, we further individually inspected the indirect effect of service value relationships between service facilities and satisfaction, as well as the indirect effect of the relationships between service facilities and WOM. Table 6 shows that the indirect effects of service value relationships between service facilities and satisfaction were higher than those of relationships between service facilities and the WOM recommendation. Thus, establishing service facilities is certainly significant. However, for the WOM recommendation of medical service by the outpatients, service value and satisfaction with the hospital must be improved.

## 5. Discussion

### 5.1. Conclusions

Service personnel significantly affected both service value and satisfaction in the medical service industry, suggesting that in a service encounter between outpatients and service personnel, a high-level service personnel provides a high service value and high satisfaction. Physical facilities significantly and positively affected service value, indicating that in a service encounter between outpatients and facilities, high-level facilities lead to a high service value. Facilities did not significantly and positively affect satisfaction; however, they influenced satisfaction through service value. This study’s finding indicated that facilities did not directly influence satisfaction. However, they indirectly and positively influenced satisfaction through mediating service value improvement variables. This study’s finding implied that if hospitals intend to strengthen outpatient satisfaction, they should begin by improving service value, which will help to improve the overall satisfaction of patients.

Service value significantly and positively influenced satisfaction, implying that a high service value leads to high outpatient satisfaction. Chang et al. [29] conducted research on healthcare systems and reported that in a service encounter with any affected patient, the LISREL model can confirm that medical staff, nursing staff, service personnel, and space equipment are effective predictors of medical treatment satisfaction. Chang and Weng [63] also used the LISREL model for verification. These studies have revealed the relevance of the relationship between service encounter factors and medical treatment satisfaction. This implies that patients select hospitals for healthcare services, and high values of attitude, environment, health, and education variables lead to high outpatient treatment satisfaction. The service encounter concept and patient satisfaction significantly and positively affected both the direct and indirect effects. These findings confirmed the results of Chang and Weng [63]. Therefore, we report that service encounter positively affects satisfaction. This study indicates that the relationships among service value, satisfaction, and WOM intention have a significant positive impact. This study’s finding implies that high service value and satisfaction lead to high WOM. Theoretically, this study fills a void in the healthcare literature. This study’s findings show a mediation effect of service value and satisfaction in the relationship between service encounters and WOM. Therefore, the medical service industry can no longer meet the needs of the patients by strengthening the number of medical services. Instead, it should focus more on medical services itself and enhance the service value through obtaining the medical identity from patients.

### 5.2. Managerial Implications

The results provide valuable strategic implications for hospitals that are seeking to establish outpatient relationships. The results also have crucial implications for marketing personnel and managers.

First, to strengthen WOM, touching consumers’ hearts is necessary. When patients perceive that a medical treatment has a high quality, they spread WOM messages to relatives, friends, and colleagues; thus, it is a crucial cue. Therefore, hospitals should effectively use their existing resources and fully understand the advantages and disadvantages of such resources. Hospitals can establish a favorable interaction with outpatients through the attitude of service personnel and by offering convenient facilities. This implies that both service value and satisfaction are key mediating variables and crucial factors to be used for enhancing the antecedent variables of WOM intention for patients in a service encounter (i.e., service personnel and physical facilities).

Enhancing patients’ overall evaluation of a hospital is a crucial prerequisite. For example, establishing a favorable doctor–patient relationship can lead to patient satisfaction and loyalty. Because of the deterioration of the doctor–patient relationship, hospital organizations must work to develop professionalism skills among service personnel, such as regular education and training for healthcare or service personnel and strengthening a sense of healthcare trust among patients. Adopting humane facilities and helping patients conveniently use medical facilities will eventually lead to more WOM recommendations. Optimally implementing these suggestions will improve hospital service value and patient satisfaction and deliver sound patient-oriented services.

Second, this study determined a significant and positive effect of the service encounter concept on patient satisfaction. Thus, service encounter aspects (service personnel and facilities) can directly increase both outpatient service value and satisfaction. Moreover, the strength of facilities indirectly enhances satisfaction through service value. When adapting to outpatients in terms of costs (money, time, efforts, and energy) incurred for obtaining service value management, improving service personnel and facilities are key factors. To strengthen patients’ satisfaction with hospital services, service personnel must strengthen service quality to provide optimal services to patients and enhance their WOM recommendation intention.

Finally, establishing national health insurance and instituting insurance restrictions and volume growth of services may not be favorable. Scholars have studied price differences and confirmed that price differences include transmission through new value. People can, through the value of their options, bring appropriateness to the forefront and focus on supporting the government’s policy and legal basis for creating consumer demand for the use of value through enhancing customer satisfaction in exchange for the delivery of new benefits. This study also determines that the overall medical service industry in Taiwan must shift its main focus from being supplier-oriented to user-oriented. To achieve this, medical institutions must develop market-oriented services. This finding supports that of a previous study and the similar viewpoints of Yoon, Choi, and Park [51]. Medical institutions must thus focus on providing customer-oriented services and enhancing their perceived service value, which is a crucial factor.

Previous studies have explored the relationship between customers and service providers who often use the satisfaction–loyalty paradigm for fundamentally approaching customer decision making. This study analyzed the determinants of customer WOM resulting from service personnel and facility perspectives to identify which perspective acts as the decisive factor influencing outpatients’ perception of service value and satisfaction with service providers. Furthermore, this study applies service value and satisfaction as the mediators between service encounter and WOM in maintaining the relationship.

### 5.3. Limitations and Future Research Directions

This study has three main limitations. The first is the problem of external validity; that is, the ability to generalize the results to countries other than Taiwan. Second, this study only focuses on the medical industry, and generalizing the conclusions of this study to other types of industries may lead to a margin of error. Third, the sampling method used in this study was convenience sampling, which was not scientifically designed. Therefore, considerable effort should be devoted to detecting any potential biases in these nonrandom samples. This study indicates that the constructs of service encounter, service value, and satisfaction are crucial antecedents of WOM intention.

These results emphasize the relevance of outpatient service in choices made in a hospital context. Future studies should consider the following recommendations. First, despite the model’s goodness of fit validated using all the analyzed indicators, empirical evidence suggests that the WOM intention is perhaps explained by additional constructs apart from service encounter, service value, and satisfaction. Future studies should address these other variables including service process, trust, commitment, altruistic behavior, and self-strengthened variables. Extending the proposed model can help in further understanding patients’ WOM intention. In addition, future researchers can apply this proposed model to understand internal marketing issues amongst hospital staff, and how to establish customer service behaviour [64,65]. In terms of organizational issues, subsequent studies can examine these variables. For example, enterprise agility, human performance, work compatibility, and organizational citizenship behavior [64,66,67,68].

Second, our analysis was based on cross-sectional data. To provide a more convincing case for causal interpretations of variable correlations, additional longitudinal studies must be conducted, and in such studies, exogenous factors can be captured before the data about endogenous criteria are collected. Third, the implementation of the National Health Insurance program and rapid expansion of large hospitals and healthcare facilities have increased the degree of competition in the Taiwanese market. Hospital movement toward enterprise management, in addition to the payment of health insurance for performance, is required; however, developing patient “self-paid” services in the healthcare market is crucial. Because the market is highly competitive, hospitals providing self-paid services to patients can gain a competitive advantage. Future researchers can grasp the market trends and people’s needs and design a precise empirical framework for developing the healthcare market and ensure its compatibility with the current health policy and market thinking in Taiwan. Fourth, this study investigates the WOM behaviors of patients in hospitals from service encounter perspectives. However, this study did not consider the other control variables (e.g., individual characteristics, demographic variables), and future research can investigate the mentioned characteristics. Fifth, this study only investigated a regional teaching hospital in Yi-Lan, Taiwan. This study suggests that future researchers may investigate different types of hospital (e.g., medical center, district hospital) to analyze the similarities and differences among different outpatients. Future researchers can apply this model to study the users of other forums or discussion areas. Finally, this study suggests that future researchers may investigate different countries to analyze the similarities and differences among different hospital outpatients.

## 6. Conclusions

This study’s conclusion was mainly based on the findings obtained using the estimated structural equation model. The empirical results suggested that all the four variables directly or indirectly affected outpatient WOM intentions. Satisfaction was determined to be the most effective predictor, followed by service personnel and service value in the indirect path. In addition, the interdependence among the four variables was confirmed for improving the model fit. The effects of the four constructs on outpatient WOM were thus high.

## Figures and Tables

**Table 1:** Demographics of respondents.

Variables	Frequency	Percentage
Gender		
Male	129	36.9
Female	221	63.1
Age		
Under 20	66	18.9
2 to 29	86	24.6
30 to 39	101	28.9
40 to 49	51	14.6
50 to 59	26	7.4
60 and older	20	5.6
First Time for Medical Treatment		
Yes	59	16.9
No	291	83.1
Division		
General Internal Medicine	71	20.3
Gastrointestinal Medicine	32	9.1
Nephrology	12	3.4
Cardiovascular Medicine	9	2.6
Chest Medicine	14	4.0
Neurosurgery	5	1.4
Metabolism	8	2.3
Family Medicine	33	9.4
Rheumatology	8	2.3
Dermatology	4	1.1
Psychiatry	6	1.7
General Surgery	32	9.1
Chest Surgery	6	1.7
Colon and Rectum Surgery	3	0.9
Cardiovascular Surgery	1	0.3
Neurological Surgery	2	0.6
Ophthalmology	5	1.4
Ear, Nose, and Throat	3	0.9
Orthopedics	10	2.9
Dental Medicine	3	0.9
Urological department	7	2.0
Obstetrics and Gynecology	31	8.9
Pediatrics	34	9.6
Rehabilitation Medicine	3	0.9
Other Division	8	2.3
Outpatient Services Time		
Morning	117	33.4
Afternoon	168	48.0
Night	65	18.6
Register Mode		
Speech Sounds	43	12.3
Internet	28	8.0
Telephone	35	10.0
Locality	82	23.4
A Diagnosis Physician	27	7.7
Registers Locality on that Day	129	36.9
Nursing Stand	1	0.3
Other	5	1.4
Medical Treatment Factor		
Relatives and Friends to Recommend	70	15.1
Traffic Convenient	86	18.5
Close to House	136	29.2
Good Facilities	42	9.0
Good Reputation	33	7.1
Service Attitude	20	4.3
Personal Habits	32	6.9
Medical Skill	21	4.5
Other	25	5.4

**Table 2:** Analysis of convergent validity.

Factors		Mean	S.D.	Composite Reliability	Average Variance Extracted	Cronbach’s Alpha
Service Encounter	Service Personnel	3.601	0.623	0.880	0.521	0.831
	Facilities	3.571	0.616	0.940	0.724	0.797
Service Value		3.369	0.740	0.934	0.718	0.904
Satisfaction		3.542	0.503	0.933	0.602	0.871
WOM		3.582	0.688	0.953	0.792	0.915

**Table 3:** Correlation matrix for measurement scales.

Constructs	SP	FAC	SV	SAT	WOM
Service Personnel	0.722				
Facilities	0.524	0.851			
Service Value	0.555	0.534	0.847		
Satisfaction	0.469	0.446	0.543	0.776	
Word-of-mouth	0.419	0.326	0.524	0.728	0.890

Note: Diagonal elements are the square roots of the average variance extracted.

**Table 4:** Model fit.

Fit Indices	χ^2^/df *	CFI **	GFI **	AGFI **	RMSEA ***
Initial research model	3.756	0.870	0.878	0.840	0.088
Modification research model	2.042	0.919	0.912	0.923	0.043
Level of acceptable fit	<3	>0.9	>0.9	>0.9	<0.08
Acceptability	Acceptable	Acceptable	Acceptable	Acceptable	Acceptable

Note: CFI = comparative fit index; GFI = Goodness of Fit Index; AGFI = Adjusted GFI; RMSEA = Root mean square error of approximation. Values indicate a fair fit. * Value of χ^2^/df below 3; ** Values of CFI, GFI, and AGFI of more than 9; *** Values of RMSEA below 0.08.

**Table 5:** Path coefficient estimates.

Latent Constructs Path	Hypothesized Model		Trimmed Model		T-Value	Results
	Path Estimate	Standard Solutions	Path Estimate	Standard Solutions		
Service Personnel→Service Value (**H1**)	0.481 *	0.423	0.578 *	0.461	5.53	Supported
Facilities→Service Value (**H2**)	0.427 *	0.304	0.390 *	0.279	3.86	Supported
Service Personnel→Satisfaction (**H3**)	0.274 *	0.299	0.294 *	0.291	3.45	Supported
Facilities→Satisfaction (**H4**)	0.113	0.118	－	－	1.34	Not Supported
Service Value→Satisfaction (**H5**)	0.261 *	0.323	0.255 *	0.316	4.38	Supported
Service Value→WOM (**H6**)	0.129 *	0.113	0.132 *	0.117	2.08	Supported
Satisfaction→WOM (**H7**)	0.869 *	0.734	0.871 *	0.734	8.13	Supported

* Significant at 0.05 level.

**Table 6:** Total effect analysis of service value.

Variable	Direct Effect	Indirect Effect	Total Effect	Ranking of Total Effect
FA-SV-SA	0.118	0.294 × 0.117 = 0.034	0.152	1
FA-SV-WOM	-	0.294 × 0.316 = 0.093	0.093	2

Note: FA = Facilities; SV = Service Value; SA = Satisfaction; WOM = Word of Mouth.
